# Comparison of Early Effectiveness of Three Different Intervention Methods in Patients with Chronic Orofacial Pain: A Randomized, Controlled Clinical Trial

**DOI:** 10.1155/2019/7954291

**Published:** 2019-03-11

**Authors:** Bartosz Dalewski, Agata Kamińska, Michał Szydłowski, Małgorzata Kozak, Ewa Sobolewska

**Affiliations:** ^1^Chair and Department of Dental Prosthetics, Pomeranian Medical University, Szczecin, Poland; ^2^Chair and Department of Dental Prosthetics, Specialists' Dental Clinic, Pomeranian Medical University, Szczecin, Poland; ^3^Faculty of Mechanical Engineering and Mechatronics, West Pomeranian University of Technology, Szczecin, Poland

## Abstract

**Background:**

Occlusal appliances are still widely used instruments in the management of orofacial pain in dentistry, yet alone or as a part of multimodal therapy. However, some of those modalities have been lacking thorough randomized assessment, and there is a conflicting evidence available. It is hypothesized that pain symptoms might improve faster and in more tangible way due to combined therapy. Also, to our best knowledge, nimesulide was never examined in this aspect, too.

**Objective:**

The aim of this study was to compare early effectiveness of routine intervention methods in patients with myofascial pain (MP) after 3 weeks' notice. Three modalities were evaluated: occlusal appliance (OA) with nonsteroidal anti-inflammatory drug (NSAID) therapy (nimesulide), occlusal appliance with dry needling (DN), and occlusal appliance (OA-control group) therapy.

**Design:**

Randomized controlled clinical trial (RCT) in which ninety patients with MP, who met the inclusion criteria, were randomly assigned to one of the three treatment groups. Sealed, opaque envelopes were used.

**Methods:**

For evaluation, each patient completed a Visual Analogue Scale (VAS) and Sleep and Pain Activity Questionnaire (SPAQ) twice, first at the beginning of the study and again after 3 weeks (0–3).

**Results:**

Posttreatment test comparison between the control group and both treated groups reveal significant differences between the control and the NSAID + occlusal appliance groups. There were also differences reported between the control and the DN + occlusal appliance groups, but these differences were, however, not statistically significant.

**Conclusions:**

Occlusal appliances in conjunction with NSAID showed better orofacial pain relief after 3 weeks of therapy, compared to the use of occlusal appliances alone or in conjunction with dry needling. Additionally, differences between pain perception and quality of life between OA and DN + OA groups were not found to be statistically significant.

## 1. Introduction

Temporomandibular disorder (TMD) is a mutual term embracing numerous health issues that involve the temporomandibular joint (TMJ), masticatory muscles, or both [1]. Its aetiology has been accepted as multifactorial [2], including personality traits, stress and psychological factors [3–5], anatomy and dental occlusion, and history of trauma resulting in internal derangement of the TMJ [6, 7]. Masticatory muscle fatigue upon awakening, muscle weakness, pain, and headaches are the most frequent symptoms of patients with TMD [8]. The prevalence of TMD ranges from 5 to 12% in general populations [9], and based on most recent estimates, approximately 65% of affected patients suffer from orofacial pain or will experience it over time [10]. According to the Subcommittee on Taxonomy of the International Association for the Study of Pain, pain is defined as a subjective sensation which is individual and depends on numerous contributing factors [1–3, 9–11]. As new versions of NSAIDs are becoming available, with more and better trials being performed, an updated evidence for their efficacy, safety, and possible adverse effects is needed for commissioners, prescribers, and consumers to make informed choices about their use. On the other hand, occlusal appliances are still commonly used instruments in the management of orofacial pain in dentistry and can be used in conjunction with NSAIDs or DN. In most of these cases, ibuprofen for 14 days is a first-line recommendation, nonetheless might be unsuitable for elderly patients with, e.g., cardiovascular complications or renal impairment [1, 2]. Some of those modalities have been lacking thorough randomized assessment, and there is a conflicting evidence available. Also, to our best knowledge, nimesulide was never analyzed in orofacial pain patients; yet, it is considered safer for long-term use in patients with comorbidities of cardiac, renal, or hepatological origin. While an occlusal appliance has been thoroughly proven in management of TMD-related pain conditions by most dental researchers and practitioners [1–3, 6–8], still it is hypothesized that symptoms might improve faster and in more tangible way due to combined therapy with NSAIDs or DN. Therefore, our goal was to determine which of these treatment options involving occlusal appliances grant significant pain relief after 3 weeks and whether it influences quality of life and sleep comfort [10].

Three modalities were evaluated: occlusal appliance with nonsteroid anti-inflammatory drug therapy (OA + NSAID group), occlusal appliance with dry needling (OA + DN group), and occlusal appliance therapy only (control group). Hence, qualitative and quantitative pain measurement options turned out to be contradictory and of limited clinical value in scientific data assessment and as such are still subject to validation [11]. For this reason, we used the VAS and SPAQ to assess pain in our study.

## 2. Materials and Methods

The protocol for this single-center clinical trial is registered with NCT03400462. The study was endorsed by the Bioethics Committee of Pomeranian Medical University in Szczecin (approval number KB-0012/83/16) and was conducted in accordance with the Declaration of Helsinki and Good Clinical Practice set forth by the International Conference on Harmonization of Technical Requirements for Registration of Pharmaceuticals for Human Use (ICH). We also ensured that the study conformed to applicable international regulatory authority laws, regulations, and guidelines.

### 2.1. Study Design and Randomization

This study was a randomized controlled clinical trial (RCT). Among patients reporting to the Prosthetic Outpatient Clinic of Pomeranian Medical University, ninety patients with myofascial pain in the preauricular area were selected. Patients who met the inclusion criteria were randomly assigned to one of the three treatment groups. One examiner performed all clinical examination, splint therapy, and dry needling and controlled the visits of all patients. Another operator, blinded to patients group assignments, performed data acquisition throughout control appointments. The recruitment period lasted from 1st July 2016 till 1st December 2017. Sealed, opaque envelopes were used for randomization as well as for achieving equal number of patients in each group.

### 2.2. Participant Selection

Inclusion criteria include patients with unilateral pain localized in the TMJ or in the preauricular area, who had no analgesic treatment in the area of the head and neck during the last 12 months, aged 18–65 years, who and had no tooth losses within occlusal support zones.

Exclusion criteria include bilateral pain, inflammation in the oral cavity that emerged as myospasm or preventive muscle contraction, earlier splint therapy, pharmacotherapy (e.g., oral contraception, hormone replacement therapy, and antidepressants), systemic diseases (e.g., rheumatic and metabolic diseases), lack of stability in the masticatory organ motor system, masticatory organ injury, pregnancy, patients undergoing orthodontic treatment, other types of inflammation in the oral cavity (e.g., pulp inflammation or impacted molars), and fibromyalgia [12].

### 2.3. Group Overview

This randomized controlled clinical trial included 2 tested groups and a control group of 30 patients each as follows: occlusal appliance (OA) with nonsteroid anti-inflammatory drug (NSAID) therapy (nimesulide), occlusal appliance with dry needling (DN), and occlusal appliance therapy (OA-control group). Participants who met the inclusion criteria completed the Sleep and Pain Activity Questionnaire (SPAQ) twice, first at the beginning of the study and again after 3 weeks of therapy. Groups consisted mostly of women ranging in age from 18 to 65 years old (mean age = 30.73).  Table 1 shows the mean ages of the tested groups by sex, age, and gender.

### 2.4. Methods of Pain-Level Evaluation

**Figure 1:**
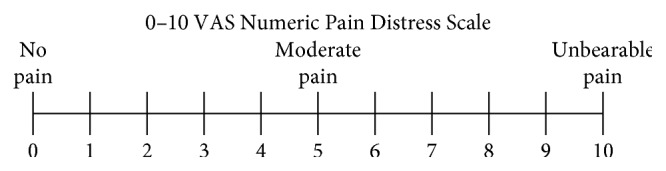
VAS example. Source: http://trialdatasolutions.com.

**Figure 2:**
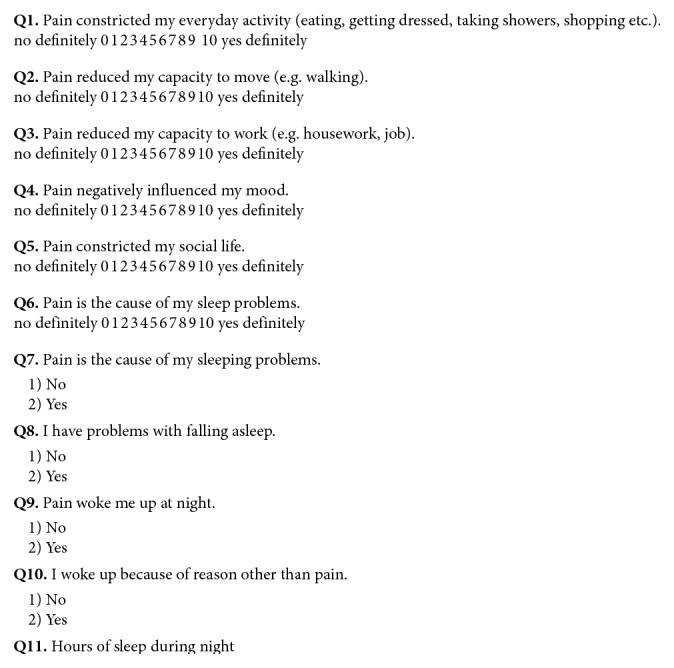
Sleep and Pain Activity Questionnaire (SPAQ). Source: own.

For evaluation, each patient completed the Visual Analogue Scale (VAS) (Figure 1) and Sleep and Pain Activity Questionnaire (SPAQ) (Figure 2) twice, first at the beginning of the study and again after 3 weeks of therapy (0–3).

#### 2.4.1. Visual Analogue Scale

This is a type of linear scale for the subjective characterization of pain. The patient describes his/her pain intensity as none, mild, moderate, or severe (Figure 1). It is an instrument which measures the subjective opinion of patient's pain. The patient describes his/her level of pain by indicating a position along a continuous line between two endpoints from 0 to 10.

Recommended VAS interpretation: no pain (0–4 mm), mild pain (5–44 mm), moderate pain (45–74 mm), and severe pain (75–100 mm) [13].

#### 2.4.2. Sleep and Pain Activity Questionnaire

Sleep and Pain Activity Questionnaire comprised Visual Analogue Scale of Pain (Figure 1) and directional questions (Figure 2). Patients were instructed to respond for questions 1–6 in accordance with VAS. For questions 7–10, only yes/no answer was possible. Question 11 was the time of sleep during night.

### 2.5. Treatment Methods

#### 2.5.1. Dry Needling

**Figure 3:**
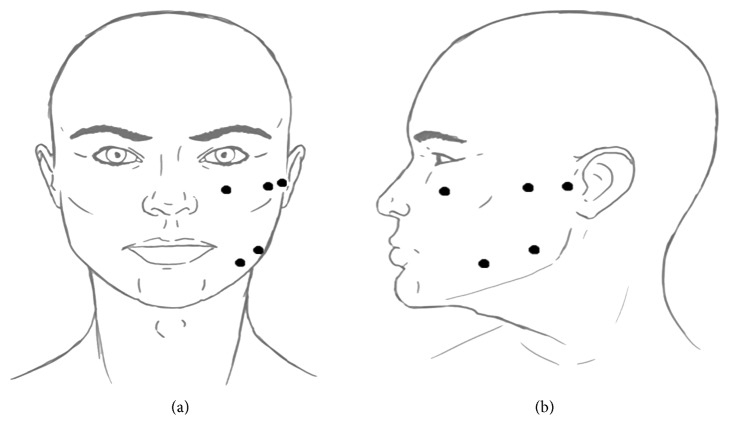
Dry needling points of insertion. Source: own.

Dry needling is a therapeutic method in which needles can be inserted into, e.g., muscles, ligaments, or scar tissue (into the myofascial trigger points) for the purpose of reducing pain. Myofascial trigger points are defined as tender nodules inside the muscle that contain hyperalgesic areas [14]. This method has been in use since 1820, and it is based on the principles of evidence-based medicine (EBM) [15]. It should not be equated to Chinese acupuncture because dry needling does not focus on energy movements, etc. Three visits were needed for this treatment modality. Visits schedule: first visit-day 1, second visit-7 days after the first, and third visit-7 days after the second. Equipment: acupuncture needle of dimensions 0.6 × 13 mm (Dragon Medical Device Ltd., China), solution for disinfection of skin (Octenisept, Schülke and Mayr GmbH), and sterile gauze 5 × 5 cm (Mato, Poland). Exposition time: 30 minutes once a week. Points of needling are presented in Figure 3.

#### 2.5.2. Splint Therapy

Splint therapy is a well-described and efficacious treatment method for TMD patients, e.g., patients with retrodiscitis and patients with muscle pain disorders such as local muscle soreness or chronic myalgia [1]. The occlusal appliance used in this study was a removable device for the maxillary arch, made of hard acrylic. The appliance was fitted over the occlusal and incisal surfaces of the teeth and precisely placed in contact with the teeth of the opposing arch. It provided canine disocclusion of the posterior teeth during eccentric movements. The patients were instructed to use the appliance at night time. Patients were made to return after 7 days for a control visit [2].

#### 2.5.3. NSAIDs

Nimesulide has anti-inflammatory and analgesic properties. Like other NSAIDs, it inhibits the action of COX. Without the further synthesis of prostaglandins, there is no factor available to excite local nociceptors. In light of this, the drug must be taken regularly for a minimum of 2 weeks to achieve appropriate blood concentrations. Dosing instructions for NSAID use are as follows: nimesulide 2 × 100 mg/24 h, i.e., one 100 mg pill twice a day for 14 days, which is the most frequently described duration of therapy in myofascial pain control and management [1, 2, 16, 17]. Patients were instructed not to use any other forms of treatment than prescribed. Each patient signed a written consent to avoid any other self-treatment throughout the duration of the study.

## 3. Results

Table 2 shows the analysis of VAS and Sleep and Pain Activity Questionnaire for each question and group. According to VAS interpretation, patients' response was categorized into four main categories: 0 (no pain), 1-2 (mild pain), 3–6 (moderate pain), and 7–10 (severe pain).

The null hypothesis was that sample difference comes from a distribution with zero median. The responses, pretreatment and posttreatment of each group, were tested using the Wilcoxon signed-rank test. The same test was used to determine the difference between groups, separately for pretreatment and posttreatment answers.  Table 3 shows the *p* values of the Wilcoxon signed-rank test at the significance level *α* = 0.05 for each question in all test groups.

**Figure 4:**
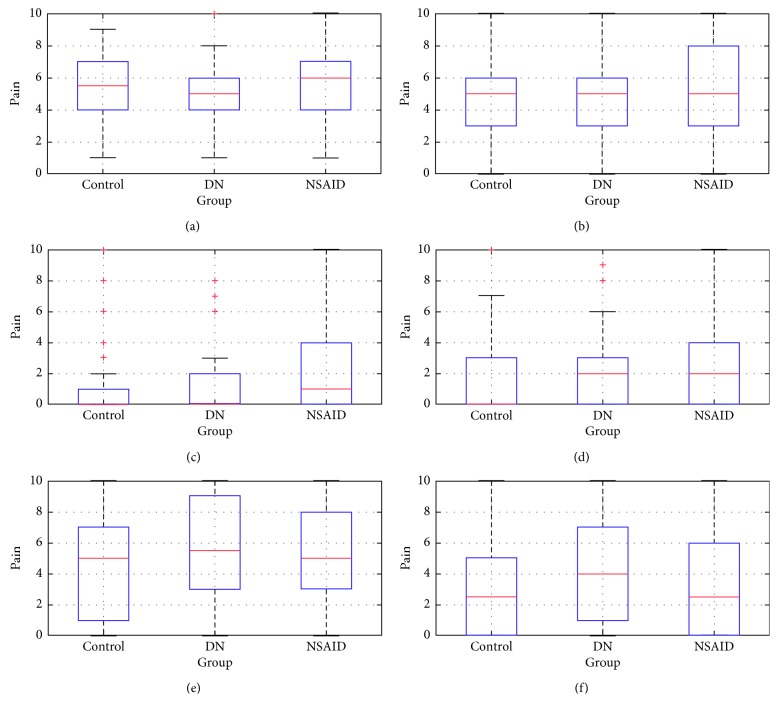
Box plots of responses of each VAS question in pretreatment test for all groups. Source: own. (a) Question 1. (b) Question 2. (c) Question 3. (d) Question 4. (e) Question 5.

All groups picture significant differences in almost all of the VAS questions (from 1 to 6) when comparing pre- vs posttreatment results. The exception is question 3 where answers show no difference in the DN-treated group (*p* value = 0.0781 at significance level 0.05). Comparison of pain intensity between control group and both treated groups results in the pretreatment stage shows no significant difference. This states that entry pain intensity levels were comparable. Results are presented at Figure 4.

**Figure 5:**
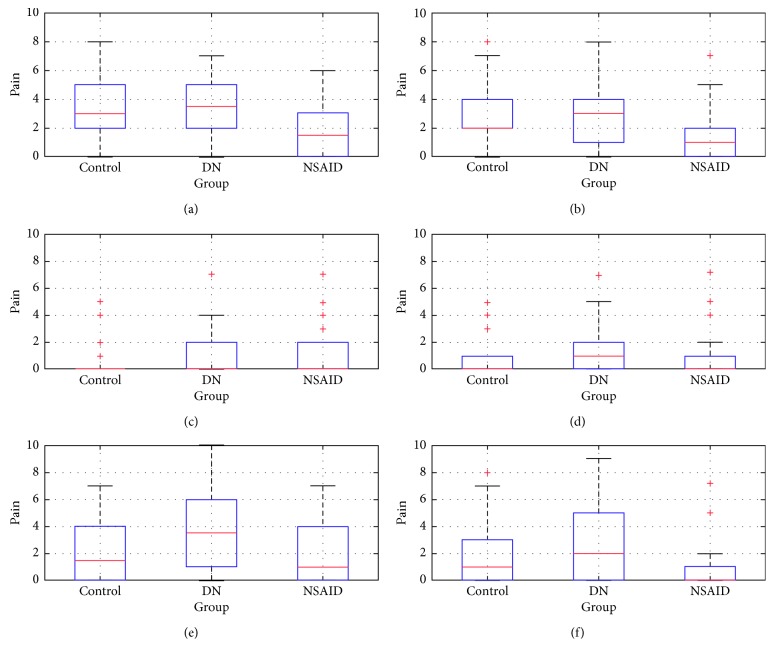
Box plots of responses of each VAS question in posttreatment test for all groups. Source: own. (a) Question 1. (b) Question 2. (c) Question 3. (d) Question 4. (e) Question 5.

Posttreatment test shows significant differences between control and NSAID-treated groups. *p* values connected to questions 1 and 2 are below the significance level *α* of the Wilcoxon signed-rank test set at 0.05. There are also significant differences between answers in questions 1, 2, 5, and 6 when comparing both treatment methods. The central mark in the box of responses indicates the median, and the bottom and top edges of the box indicate the first and third quartile, respectively. The whiskers extend to the most extreme data points not considering outliers, and the outliers were plotted individually using the plus “+” symbol. Results are presented in Figure 5.

Analysis of the SPAQ shows significance difference in answers among the groups in the pre-post comparison. For cases where the answer count was 5 and more, the chi-square test was computed, and in other cases, Fisher's exact test was evaluated. The results are presented in Table 4.

According to the control group, answers to questions from 7 to 10 differ significantly. As well as answers to questions 7, 8, and 9 in the NSAID group (M1) and the DN group (M2) revealed significantly different answers only to questions 7 and 9, when comparing pretreatment and posttreatment responses.

Comparison of both examined groups with controls in terms of posttreatment responses regarding question 7 to 10 showed no significant difference. Detailed analysis was performed by using Fisher's exact test while the count in some of categories was less than 5. The results are presented in Table 5.

All groups presented significant differences according to time of sleep in pre-/posttreatment comparison. Assessment of the control group and the NSAID group (M1) in posttreatment shows significance in the Wilcoxon signed-rank test. The results are shown in Table 6.

The average sleep time is presented in Table 7 Also differences in pain perception and quality of life between OA and OA+DN groups were statistically insignificant.

## 4. Discussion

Nimesulide is marketed in more than 50 countries. Yet, to the best of our knowledge, this is the first report, evaluating its usage in TMD patients. From our findings, the OA + NSAID group showed greater short-term improvements in myofascial pain located in the preauricular area compared to the OA + DN and OA only (control) groups. These differences were found to be statistically significant while the OA + DN group was not found to be superior over OA only (control group). In addition, statistically important improvement in sleep quality of OP patients was also noted in the OA + NSAID group. Bocanegra et al. evaluated patients with moderate to severe pain after extraction of impacted third molars. In their study, nimesulide and ibuprofen provided effective pain control in first 24 hours after surgery. They concluded that, despite both medicaments were well tolerated, the therapeutic effect of nimesulide had a faster, less than 15 minutes onset, and was stronger (according to patients' opinion) than ibuprofen [18]. In different work, nimesulide was found to be more effective in relieving pain in osteoarthritis of the hip and knees and with faster onset of action and less side effects than diclofenac and celecoxib [16, 17, 19]. It also showed better postoperative pain relief compared to ibuprofen, having a faster analgesic effect (<15 minutes) and a better patient rating of effectiveness compared to those receiving ibuprofen [20]. Hence, two different groups of clinicians may be involved in the management of orofacial pain, i.e., pain physicians and pain-trained dentists. For physicians, methods of choice comprise usually evidence-based pharmacotherapy and more localized pain interventions such as injections and needling whereas the approach by dentists to the same problem would be a splint in conjunction with physiotherapy or evidence-based complementary methods [1, 2]. Hong described the effects of injection with a local anesthetic agent (LAA) and DN into a myofascial trigger point (TrP) of the upper trapezius muscle in a group of 58 patients. Trigger point injections with 0.5% lidocaine were administered to 26 patients (Group I), and DN was performed on TrPs in 15 patients (Group II). Improvement was assessed by measuring the subjective pain intensity, the pain threshold of the TrP, and the range of motion of the cervical spine. Statistically significant improvement occurred immediately after injection within patients of both groups. However, the group treated with DN had postinjection soreness of significantly greater intensity and longer duration than those treated with lidocaine injection. The author concluded that it is essential to elicit a “local twitch response” (LTR) during injection to obtain an immediately desirable effect. Due to his findings, TrP injection with 0.5% lidocaine is recommended, because it reduces the intensity and duration of postinjection soreness compared to that produced by dry needling [21]. In a study of parallel design, Dıraçoğlu et al. attempted to test the hypothesis that DN is more effective than sham DN in relieving myofascial pain of the temporomandibular muscles. They randomly associated fifty-two subjects with diagnosed myofascial trigger points into two groups: study group (*N*: 26) and placebo group (*N*: 26). DN was applied using acupuncture needles, whereas sham DN was administered to the placebo group. Pain pressure threshold (PPT) was measured with pressure algometry, pain intensity was rated using a 10 cm Visual Analogue Scale (VAS), and the unassisted jaw opening without pain measurement was performed. Mean algometric values were significantly higher in the study group when compared to the placebo group (*p* values less than 0.05). There were no differences between the two groups in terms of VAS and unassisted jaw opening without pain values. According to these findings, DN appears to be an effective treatment method in relieving the pain and tenderness of TrPs; however, comparison between different treatment modalities was not scrutinized [22]. Varoli et al. examined two types of NSAIDs in the management of painful TMD in a placebo-controlled study. Each patient in their work received a flat, occlusal splint with canine guidance and simultaneous occlusal contacts. They were then randomly assigned to one of the three groups: (1) NSAIDs (sodium diclofenac), (2) panacea (sodium diclofenac + carisoprodol + acetaminophen + caffeine), and (3) placebo. The intensity of pain was assessed with the use of the VAS. After data evaluation, significant differences were observed. Overall, they concluded that NSAID therapy promotes analgesia from the third day, while in the placebo group, it was achieved on the eighth day [23]. These results are consistent with our study, where the NSAID group showed faster onset and superior results over the control group. On the other hand, a controlled clinical trial published by Gonzalez-Perez et al. suggests significant efficacy of deep DN in a group of 36 patients with myofascial pain located in the external pterygoid muscle. Three sessions were performed for each patient at an interval of 1 week and clinical assessments at 2 weeks, 1 month, 2 months, and 6 months after finishing the treatment. As in most of the studies designed to evaluate TMD, the VAS was used for pain assessment. Also, the range of mandibular movements before and after intervention was examined. This study proven statistically significant relationship (*p* < 0.01) between therapeutic intervention and the improvement of pain and jaw movements, which continued up to 6 months after treatment. When pain reduction was greater, the higher was the intensity of pain at baseline. The authors concluded that further studies are needed; however, their findings suggest that deep dry needling in the trigger point in the external pterygoid muscle can be effective in the management of patients with myofascial pain located in that muscle [24]. In our study, DN did not show any evidence to be more effective than OA. Ozkan et al. compared two therapy patterns: occlusal splint vs occlusal splint + trigger point injections with local anesthetic solution of 0.5 ml lidocaine +0.5 ml saline/0.1 ml triamcinolone acetonide. The occlusal splint therapy group was instructed to wear the splint at night for a period of three months. The group receiving trigger point injections undertook three visits with two-day intervals between them. At the first and second visits, local anesthetic + saline was administered, while at the third visit, 0.1 ml triamcinolone acetonide injection was administered. They concluded that at follow-up, positive results regarding signs and symptoms were found in both groups as follows: significant reduction in the frequency of pain and intensity of pain (*p* < 0.001) and decrease of TrPs in the masticatory muscles, which was statistically significant (Group 1: *p*=0.004; Group 2: *p* < 0.001). The general outcome of the study by Ozkan et al. is that injection combined with occlusal splint therapy was far more effective in the treatment of myofascial TMD pain for the improvement of signs and symptoms, which is partly consistent with our own findings [25]. The influence of pharmacological treatment on pain intensity was also investigated by Rizzatti-Barbosa et al. Their randomized trial consisted of three treated groups: Group I: benzodiazepine, orphenadrine citrate, and occlusal splint (BOS), Group II: orphenadrine citrate, occlusal splint, and benzodiazepine (OSB), and Group III: occlusal splint, benzodiazepine, and orphenadrine citrate (SBO). Administered drugs were as follows: five mg/day of benzodiazepine, 35 mg/4-hour intervals of orphenadrine citrate. An occlusal splint with full arch coverage and no cuspid rise was used. One of the three specific protocol treatments was applied for 21 days, with the three therapeutic modalities consecutively. After 21 days of therapy, no significant differences were found among the examined groups [26], while in our study, therapeutic intervention with OA and NSAID showed significantly better results.

## 5. Conclusions

In this study, occlusal appliance in conjunction with nimesulide showed superior orofacial pain relief and improvement of sleep quality after 3 weeks of therapy in comparison with occlusal appliance used unaided or in conjunction with DN. As such, it should be considered as an NSAID of choice in the management of TMD pain, mostly due to the faster onset of action and less side effects than diclofenac, celecoxibe, and ibuprofen [16, 17, 19, 26].

## Figures and Tables

**Table 1:** Group characteristics according to sex, age, and gender.

	Control		NSAID		DN	
	Count	Ratio (%)	Count	Ratio (%)	Count	Ratio (%)
*Sex*						
Female	25	83.33	24	80.00	23	76.67
Male	5	16.67	6	20.00	7	23.33
Sum	30	100	30	100	30	100

*Age (years)*						
Max	52		56		65	
Min	18		18		21	
Mean	28.7		31.2		31.3	

**Table 2:** Answer data in simplified scale (analysis of VAS and Sleep and Pain Activity Questionnaire for each question and group).

Group		Pain before treatment								Pain after treatment							
		No (0)		Mild (1-2)		Moderate (3–6)		Severe (7–10)		No (0)		Mild (1-2)		Moderate (3–6)		Severe (7–10)	
		*n*	*f*	*n*	*f*	*n*	*f*	*n*	*f*	*n*	*f*	*n*	*f*	*n*	*f*	*n*	*f*
Q1	Control	0	0	15	0.5	5	0.17	10	0.33	3	0.1	17	0.57	8	0.27	2	0.07
	DN	0	0	20	0.67	3	0.1	7	0.23	3	0.1	20	0.67	6	0.2	1	0.03
	NSAID	0	0	20	0.67	2	0.07	8	0.27	8	0.27	8	0.27	14	0.47	0	0

Q2	Control	2	0.07	17	0.57	4	0.13	7	0.23	5	0.17	12	0.4	11	0.37	2	0.07
	DN	2	0.07	19	0.63	3	0.1	6	0.2	4	0.13	15	0.5	10	0.33	1	0.03
	NSAID	1	0.03	15	0.5	4	0.13	10	0.33	10	0.33	5	0.17	13	0.43	2	0.07

Q3	Control	22	0.73	4	0.13	2	0.07	2	0.07	24	0.8	2	0.07	4	0.13	0	0
	DN	18	0.6	2	0.07	6	0.2	4	0.13	18	0.6	3	0.1	7	0.23	2	0.07
	NSAID	14	0.47	7	0.23	5	0.17	4	0.13	21	0.7	3	0.1	5	0.17	1	0.03

Q4	Control	17	0.57	7	0.23	4	0.13	2	0.07	18	0.6	6	0.2	6	0.2	0	0
	DN	8	0.27	8	0.27	11	0.37	3	0.1	12	0.4	4	0.13	11	0.37	3	0.1
	NSAID	8	0.27	6	0.2	12	0.4	4	0.13	21	0.7	3	0.1	5	0.17	1	0.03

Q5	Control	2	0.07	11	0.37	8	0.27	9	0.3	8	0.27	10	0.33	9	0.3	3	0.1
	DN	2	0.07	12	0.4	4	0.13	12	0.4	6	0.2	12	0.4	7	0.23	5	0.17
	NSAID	2	0.07	10	0.33	4	0.13	14	0.47	13	0.43	9	0.3	6	0.2	2	0.07

Q6	Control	9	0.3	10	0.33	6	0.2	5	0.17	14	0.47	7	0.23	6	0.2	3	0.1
	DN	5	0.17	8	0.27	9	0.3	8	0.27	10	0.33	8	0.27	8	0.27	4	0.13
	NSAID	9	0.3	8	0.27	6	0.2	2	0.07	21	0.7	2	0.07	5	0.17	2	0.07

*n*, count; *f*, fraction; Q, question number in the questionnaire.

**Table 3:** *p* values of the Wilcoxon signed-rank test.

Question	CT1_vs_CT2	M1T1_vs_M1T2	M2T1_vs_M2T2
*Group pretreatment vs posttreatment*			
1	0.0001	*p* < 0.0001	*p* < 0.0001
2	0.0001	*p* < 0.0001	*p* < 0.0001
3	0.0195	*p* < 0.0001	0.0781
4	0.0059	*p* < 0.0001	0.0050
5	0.0001	*p* < 0.0001	*p* < 0.0001
6	0.0020	*p* < 0.0001	*p* < 0.0001

Question	CT1_vs_M1T1	CT1_vs_M2T1	M1T1_vs_M2T1
*Pretreatment test*			
1	0.2893	0.7238	0.3938
2	0.4052	0.8009	0.7080
3	0.1764	0.6621	0.1959
4	0.3440	0.4552	0.8304
5	0.2078	0.2259	0.8820
6	0.6560	0.2617	0.3312

Question	CT2_vs_M1T2	CT2_vs_M2T2	M1T2_vs_M2T2
*Posttreatment test*			
1	0.0035	0.8073	0.0023
2	0.0483	0.7757	0.0196
3	0.5510	0.1758	0.2192
4	0.6726	0.2272	0.1100
5	0.4637	0.0867	0.0348
6	0.1315	0.2529	0.0170

Values are significant at *α* = 0.05. CT1, control group first test; CT2, control group second test after seven days; M1T1, OA + NSAID group first test; M1T2, OA + NSAID second test after seven days; M2T1, OA + DN group first test; M2T2, OA + DN group second test after seven days; *α*, significance level.

**Table 4:** *p* values of the frequency (*P*), chi-square test values (Chi), and Fisher's exact test (*P*(*F*)) results for the control group, M1 group, and M2 group. Values are significant at *α* = 0.05 level.

		Pretreatment (number/% of yes/no answers)		Posttreatment (number/% of yes/no answers)		Chi	*P*	*P*(*F*)
*Control group*								
Q7	Yes	14	47%	4	13%	7.9365	0.0048	0.0101
	No	16	53%	26	87%			
Q8	Yes	5	17%	1	3%	2.946	0.0852	0.1945
	No	25	83%	29	97%			
Q9	Yes	8	27%	1	3%	6.4052	0.0114	0.013
	No	22	73%	29	97%			
Q10	Yes	0	0%	1	3%	—	—	—
	No	30	100%	29	97%			

*M1 group*								
Q7	Yes	16	53%	4	13%	10.8	0.001	0.0022
	No	14	47%	26	87%			
Q8	Yes	9	30%	3	10%	3.75	0.0528	0.1042
	No	21	70%	27	90%			
Q9	Yes	11	37%	3	10%	7.6073	0.0058	0.0102
	No	19	63%	27	90%			
Q10	Yes	5	17%	3	10%	0.7436	0.3885	0.67
	No	25	83%	27	90%			

*M1 group*								
Q7	Yes	16	53%	3	10%	13.016	0.0003	0.0006
	No	14	47%	27	90%			
Q8	Yes	9	30%	4	13%	2.455	0.1172	0.206
	No	21	70%	26	87%			
Q9	Yes	7	23%	3	10%	1.92	0.1659	0.1894
	No	23	77%	27	90%			
Q10	Yes	3	10%	3	10%	—	—	—
	No	27	90%	27	90%			

**Table 5:** *P*(*F*) values of Fisher's exact test. Values are significant at *α* = 0.05 level.

	CT_VS_M1	CT_VS_M2
Q	*P*(*F*)	*P*(*F*)
Q7	1	1
Q8	0.612	0.3533
Q9	1	0.612
Q10	1	0.612

**Table 6:** Pretreatment and posttreatment comparison in all groups and posttreatment effect comparison between groups: *p* values of the Wilcoxon signed-rank test. Values are significant at *α* = 0.05 level.

Q	CT1 vs CT2	M1T1 vs M1T2	M2T1 vs M2T2
*Pretreatment and posttreatment comparison*			
Q11	0.01025	0.00079	0.00049

Q	CT vs M1	CT vs M2	M1 vs M2
*Posttreatment effect comparison*			
Q11	0.0075	0.4012	0.06791

**Table 7:** Pretreatment and posttreatment sleep time mean comparison.

	Pretreatment (sleep mean)	Posttreatment (sleep mean)
Control	6.15	6.4667
M1	6.464	7.1333
M2	6.45	6.7833

## Data Availability

The data used to support the findings of this study are available from the corresponding author upon request.
